# Editorial: The epidemiology of missed and delayed medical diagnosis: implications for health equity and public health

**DOI:** 10.3389/fpubh.2025.1613896

**Published:** 2025-05-28

**Authors:** Doug Salvador, Kenneth A. Mundt

**Affiliations:** ^1^Department of Medicine, University of Massachusetts Chan Medical School - Baystate, Springfield, MA, United States; ^2^ATW Health Solutions, Chicago, IL, United States; ^3^Department of Biostatistics and Epidemiology, School of Public Health and Health Sciences, University of Massachusetts, Amherst, MA, United States

**Keywords:** diagnostic error, patient safety, epidemiology, healthcare quality, public health, health equity

## Overview of the Research Topic

The pursuit of diagnostic excellence and the reduction of diagnostic errors improve patient safety and public health. However, the broad range of factors leading to missed or delayed diagnoses at any point in time complicates the identification of and interventions on modifiable factors that reduce or prevent diagnostic errors. Although the importance of diagnostic safety and the costs in terms of human health as well as medical care expenditures are appreciated, the level of research effort and investment into identifying preventable causes of diagnostic errors has been lagging. This in part likely stems from deficiencies in defining specific types of diagnostic error and the lack of standard research approaches for identifying root causes and especially preventable ones. Medicine is practiced in an increasingly complex socio-technical system where causal relationships between system attributes and outcomes including accurate diagnosis may not be visible using traditional epidemiological methods. Additionally, the stigma surrounding missed, delayed or wrong diagnosis no doubt intimidates some practitioners, and subsequently impedes if not precludes the objective examination of all the systemic, institutional and patient factors involved in the science and art of diagnosis. Furthermore, the role of the patient in quality diagnosis is increasingly acknowledged, but how effective patient participation can be enhanced across diverse age, gender, cultural, educational and socio-economic groups remains unclear and challenging.

The epidemiology of missed and delayed diagnosis: implications for health equity and public health, is a collection of invited papers with a focus on epidemiological approaches and perspectives in improving methods leading to an understanding of the preventable causes of diagnostic error, including health equity and public health aspects. This provided a forum in which contributions from various stakeholders were intended to stimulate the exchange of ideas and scientific approaches that transcend professional niches to inspire additional epidemiological research on diagnostic excellence. As expected, the 10 published articles represent a mix of topics, study approaches, and professional perspectives that share a central theme of improving diagnoses.

Hunter et al. set the epidemiological methods stage by highlighting the challenges but also the criticality of defining suboptimal diagnoses, preferably in ways that objectively can be measured and evaluated against a range of possible risk factors from many different domains (and not simply practitioner training or performance). They discuss how diagnostic errors likely arise due to multiple coincidental “partitioning factors” including abstract factors reflecting individual behaviors, beliefs and communication barriers. They conclude that guided by well-constructed research hypotheses, critical thinking and adherence to good epidemiological methods, insights into contributors to diagnostic excellence will be identified.

Five papers, though addressing different research topics, report on original research conducted to elucidate different aspects of diagnostic error.

McDonald et al. examined data from a representative sample of US patient survey responses regarding their diagnostic experiences. Over one third of the sample reported experiencing a “diagnostic problem or mistake” in the preceding 4 years. The subgroup analyses and reported associations raise interesting questions for promising future study.

Maleki et al. explored sociodemographic inequalities in the postnatal care coverage (PNC) provided women in Iran. They noted that these disparities and the failure to deliver proper PNC to all women regardless of age educational level, region, etc., increases the risk of adverse postnatal health consequences.

During the COVID-19 experience in China, Wang et al. demonstrated that self-care practices resulting from limited access to care providers was common, although certain subgroups of the population were less inclined to maintain regular exercise and weight control routines.

Another study in China compared health self-assessment ratings of those enrolled in the Urban and Rural Residents Medical Insurance (URRMI) with those not enrolled. Yu et al. reported that those enrolled in the URRMI reported more favorable health self-assessments.

Atac et al. presented an interesting study in which family physicians estimated the probability of diagnosis in three clinical scenarios about cancers (breast, cervical, and colorectal) and three infections (pneumonia, urinary tract infection, and COVID-19). For all scenarios, physicians' estimates were higher than the evidence range.

One paper described a protocol for a planned study of improving communication about diagnosis in pediatric care. Rasooly et al. frame pediatric diagnosis as a process stemming from “systems-of-work” communication and propose methos for assessing the validity of various diagnostic error detection methods.

Syros et al. present findings from a systematic review of the literature on barriers to care experienced by musculoskeletal sarcoma patients. They defined four types of barriers to obtaining appropriate care, including socioeconomic, geographic, healthcare quality and sociocultural factors, noting that assessing these can lead to beneficial interventions to improve quality and reducing delays in obtaining care.

Two commentaries rounded out the Research Topic. Coronado-Vázquez et al. describe a model for supporting cancer prevention and early diagnosis and treatment of cancer among adults experiencing homelessness in Madrid, Athens, Vienna, and Cambridge. They concluded that the structural injustices in the health systems in these regions, including recognizing citizenship and simple “generosity” must be addressed to reduce health inequities faced by this population.

Another commentary presented a compelling argument for critically assessing the necessity and problems with using race and ethnicity in diagnostic, treatment and other clinical support tools. Using the Vaginal Birth After Cesaran (VBAC) calculator as a case study, Kimani summarized the use of racial and ethnic categories in science and medicine historically and currently, and concludes that medical algorithms based on these interfere with efforts to reduce maternal morbidity and mortality.

This Research Topic provides a wide range of perspectives and approaches to investigate and understand the important public health problem of preventable harm caused by missed and delayed diagnosis. The articles offer novel insights, methods to emulate, and applications of epidemiological tools to several populations and disease entities. Diagnostic processes and the settings where they occur are rapidly changing with more remote care, patient self-care, artificial intelligence and other advanced diagnostic technology. Future research on these systems and new methods to understand the causal relationships between process and system attributes and diagnostic safety outcomes will require better epidemiological methods that identify areas for improvement, leading to better patient and public health.

